# Simulation and Analysis of SAR Images of Oceanic Shear-Wave-Generated Eddies

**DOI:** 10.3390/s19071529

**Published:** 2019-03-29

**Authors:** Yuhang Wang, Min Yang, Jinsong Chong

**Affiliations:** 1 National Key Lab of Microwave Imaging Technology, Beijing 100190, China; wangyuhang15@mails.ucas.ac.cn (Y.W.); minyang993@126.com (M.Y.); 2 Institute of Electronics, Chinese Academy of Sciences, Beijing 100190, China; 3 School of Electronics, Electrical and Communication Engineering, University of Chinese Academy of Sciences, Beijing 101408, China

**Keywords:** oceanic eddies, shear-wave-generated eddies, burgers-Rott vortex model, SAR image simulation

## Abstract

Synthetic Aperture Radar (SAR) is widely used in oceanic eddies research. High-resolution SAR images should be useful in revealing eddy features and investigating the eddy imaging mechanism. However, SAR imaging is affected by various radar parameters and environmental factors, which makes it quite difficult to learn directly from SAR eddy images. In order to interpret and evaluate eddy images, developing a proper simulation method is necessary. However, seldom has a SAR simulation method for oceanic eddies, especially for shear-wave-generated eddies, been established. As a step forward, we propose a simulation method for oceanic shear-wave-generated eddies. The Burgers-Rott vortex model is used to specify the surface current field of the simulated eddies. Images are then simulated for a range of different radar frequencies, radar look directions, wind speeds, and wind directions. The results show that the simulated images are consistent with actual SAR images. The effects of different radar parameters and wind fields on SAR eddy imaging are analyzed by qualitative and quantitative methods. Overall, the simulated images produce a surface pattern and brightness variations with characteristics resembling actual SAR images of oceanic eddies.

## 1. Introduction

As an important part of ocean dynamics, the formation, motion, and dissipation of eddies are significant research issues for oceanographers. The movement of eddies will agitate seawater and expand the scope of biological organisms, thus affecting the distribution of organics and the transportation of heat and salt in the ocean. Synthetic Aperture Radar (SAR) is capable of acquiring high-resolution images all-day and in all weather, and it is sensitive to minor changes of ocean surface roughness produced by eddies. Therefore, SAR images are advantageous in the identification and study of oceanic eddies. At present, SAR images are widely used for the detection [1,2,3,4,5] and statistical research [6,7,8] of oceanic eddies.

SAR imaging of eddies is mainly determined by four mechanisms. The first one is the shear-wave mechanism [8,9,10,11,12], which is associated with the wave/current interactions in the zones of current shear, and the eddy manifests itself in the form of regions of modulated normalized radar cross section (NRCS) and twisted into spirals in SAR images. The second one is the film mechanism [8,9,10,13], which is caused by the suppression of gravity-capillary waves by surface films of natural origin. Such surface films alter the sea surface tension by smoothing ripples and cause the diminishing of NRCS. The third eddy manifestation mechanism is one possible due to variations in the wind field caused by changes in the atmospheric boundary layer across an oceanic temperature front [8,9,13,14]. The fourth mechanism contributes to eddy visualization in SAR images by tracing currents with visible particles, e.g., ice pieces [8,9,10,15].

Oceanic eddies are captured by SAR, 29% of which are shear-wave-generated; the rest are mostly film-generated [8]. Film generated eddies, owing to the notable smoothing effect on ocean surface roughness, are usually obvious and easy to detect. However, shear-wave-generated eddies, appearing on SAR images through surface roughness modulation from wave-current interaction, are usually less obvious. Image features of shear-wave-generated eddies are affected by various factors, such as wind, current, seafloor topography, SAR parameters, etc. Features of shear-wave-generated eddies are usually the product of several of the above factors, which are sometimes even hard to recognize. Besides, the imaging conditions of actual SAR images is usually unknown and hard to control, which makes it quite difficult to conclude feature patterns of eddies directly from SAR images. Thus, this paper proposes a simulation method whose results may provide guidance for interpreting the features of the shear-wave-generated eddies in SAR images and facilitate detection and visualization of shear-wave-generated eddies in future works.

**Figure 1:**
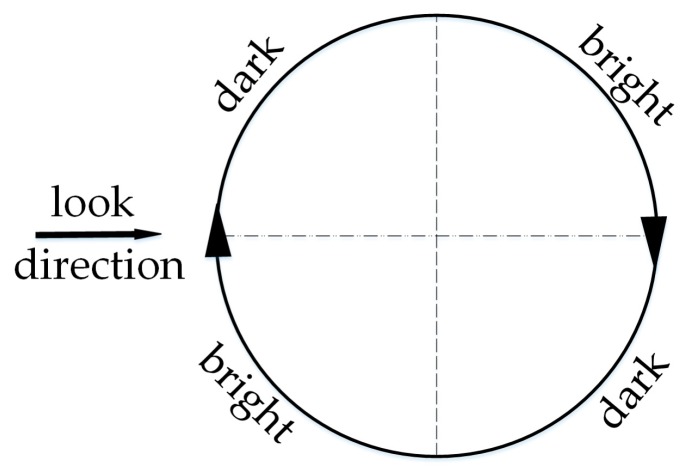
Schematic diagram of imaging geometry for idealized anticyclonic eddy [11].

Shear-wave-generated eddies in SAR images will present brightness variations which correspond to the smoothed and rough region [16]. Ref. [11] presents a schematic diagram of imaging geometry for an idealized anticyclonic eddy, as shown in Figure 1. A dark line appears on the upper left and lower right portions of the eddy, and a bright line on the lower left and upper right of the eddy. This brightness variation of the shear-wave-generated eddy depends on the radar look direction and wind field. By analyzing two airborne SAR images of the same eddy under two orthogonal flight tracks, Lyzenga [11] found that under two orthogonal radar look directions, the eddy spirals show the opposite brightness variation. Johannessen [12] also found that the changes in the radar look direction result in varying NRCS along eddy spirals. In addition, Ivanov [10] mentioned that wind speed has an effect on the eddy feature in SAR images. However, the SAR parameters used to observe the eddies are only a subset of the full parameter set. Thus, the results of these SAR images only represent some of the possible eddy observations.

In order to interpret and evaluate the effects of the radar look direction and wind field conditions on SAR eddy imaging, numerical imaging simulations can serve as an effective tool for the systematical investigation of brightness variations of shear-wave-generated eddies. Cooper et al. [16] used the inertial instability model and the Environmental Research Institute of Michigan (ERIM) Ocean Model to simulate SAR images of film-generated eddies and analyzed the effects of radar parameters and wind fields on eddy imaging. However, SAR image simulation for shear-wave-generated eddies has rarely been performed, and the influence of different radar parameters and wind field conditions on imaging characteristics of these eddies has not been discussed systematically. Therefore, the objective of the present study is to propose a simulation method to simulate radar backscatter images for shear-wave-generated eddies and analyze the influence of the radar look direction and wind field conditions on SAR eddy imaging.

The remainder of this paper is organized as follows: In Section 2, the simulation method and the model used is described in detail. Section 3 verifies the correctness of the proposed method based on ENVISAT-1 ASAR and ERS-2 SAR images. The influence of different radar looks directions, wind directions and wind speeds on SAR eddy imaging are discussed in Section 4. We conclude with a discussion of the applicability of our research for future investigations on shear-wave-generated eddies in Section 5.

## 2. Simulation Method of SAR Images of Oceanic Shear-Wave-Generated Eddies

The simulation process was divided into two steps: Firstly, the current field of eddy was established on the basis of the Burgers-Rott vortex model. Then, the established current field of the eddy and sea surface wind field were inputted into an oceanic SAR imaging simulation model, and the simulated image was obtained.

### 2.1. Establishment of Surface Current Field

The features of shear-wave-generated eddies mainly present themselves as spirals in SAR images. Therefore, the hydrodynamic vortex model which presents the eddy spirals should be used to establish the eddy current field. Typical vortex models which are qualified include the Rankine vortex [17], Oseen vortex [18], Burgers-Rott vortex [19,20,21,22], and Sullivan vortex [23]. The Rankine vortex model assumes that the vorticity of its core is discontinuous, which is not the case for shear-wave-generated eddies. The Oseen vortex model is hypothesized to be a plane flow; however, shear-wave-generated eddies should be three-dimensional. Therefore, two three-dimensional vortex models with an extra axial velocity have been proposed, i.e., the Burgers-Rott and the Sullivan vortex models. The Burgers-Rott vortex model is a spiral vortex instead of a two-cell vortex like that of the Sullivan vortex model. Due to the fact that the features of shear-wave-generated eddies mainly present as spirals in SAR images, the Burgers-Rott vortex model which can present the eddy spirals is most qualified to establish the current field of the shear-wave-generated eddy.

The Burgers-Rott vortex model is actually an exact solution obtained from the Navier-Stokes equation with the assumption that the vortex is stationary and axisymmetric, and the radial velocity $V_{r}$ and the circumferential velocity $V_{\theta }$ are independent of the axial coordinate z [19,20,21,22], i.e.,

(1) $$V_{r}=V_{r}(r),\;V_{\theta }=V_{\theta }(r),\;V_{z}=V_{z}(r,z)$$

The continuity equation of the incompressible flow is

(2) $$\frac{\partial (rV_{r})}{\partial r}+\frac{\partial (rV_{z})}{\partial z}=0$$

The projection of the N-S equation in the circumferential direction can be expressed as

(3) $$V_{r}\frac{\partial V_{\theta }}{\partial r}+\frac{V_{r}V_{\theta }}{r}=\upsilon (\frac{\partial^{2}V_{\theta }}{\partial r^{2}}+\frac{1}{r}\frac{\partial V_{\theta }}{\partial r}-\frac{V_{\theta }}{r^{2}})$$

where υ is the kinematic viscosity coefficient of the molecule. Providing the circulation of velocity $\Gamma =2\pi rV_{\theta }$, Equation (3) can be rewritten as

(4) $$\frac{d^{2}\Gamma }{dr^{2}}=(\frac{1}{r}+\frac{V_{r}}{\upsilon })\frac{d\Gamma }{dr}$$

Since the radial velocity $V_{r}$ is only a function of the radial coordinate *r*, the general solution of Equation (4) can be expressed as

(5) $$\Gamma =c_{1}H(r)+c_{2}$$

where $c_{1}$ and $c_{1}$ are constants, and

(6) $$H(r)=\int_{0}^{r}x\exp\left\{\int_{0}^{x}\left[V_{r}(s)/\upsilon \right]ds\right\}dx$$

Considering r→0 at the vortex core, Γ→0 should be ensured. Consequently, we know that $c_{2}=0$. At r→∞, $\Gamma \to \Gamma_{0}$, we can obtain $c_{1}=\Gamma_{0}/H(\infty )$. Following this, the circumferential velocity $V_{\theta }$ can be computed as

(7) $$V_{\theta }=\frac{H(r)}{2\pi rH(\infty )}\Gamma_{0}$$

To determine the unknown relationship between the axial velocity $V_{z}$, the radial velocity $V_{r}$ and *r*, it is assumed that the $V_{z}$ is independent of the radial coordinate *r* and has a linear relationship with *z*, i.e.,

(8) $$V_{z}=\alpha z,\;\alpha >0$$

where α is a constant named suction intensity. Substituting Equation (8) into Equation (2), $d(rV_{r})/dr=-\alpha r$ can be obtained, and the general solution is $V_{r}=-\frac{\alpha }{2}r+c/r$. Considering r→0 at the vortex core, a finite velocity is ensured. Therefore, the integral constant c is zero, and

(9) $$V_{r}=-\frac{\alpha }{2}r$$

By substituting Equation (9) into Equation (6), H(r) can be obtained. Following this, the circumferential velocity $V_{\theta }$ can be computed as

(10) $$V_{\theta }=\frac{\Gamma_{0}}{2\pi r}\left[1-\exp(-\frac{\alpha r^{2}}{4\upsilon })\right]$$

According to the above derivation, the three-dimensional velocity field of the vortex in the cylindrical coordinate system is given by Equations (8)–(10). Transform Equations (9) and (10) into the Cartesian rectangular coordinate, and the vortex velocity field can be expressed as

(11) $$\begin{array}{l} V_{x}=-\frac{\alpha }{2}x-\frac{\Gamma_{0}\alpha }{8\pi \upsilon }y\\ V_{y}=\frac{\Gamma_{0}\alpha }{8\pi \upsilon }x-\frac{\alpha }{2}y \end{array}$$

where $V_{x}$ is the component of the velocity field in the *x* direction, and *V_y_* is the component of the velocity field in the y direction. Therefore, by setting the parameter values of α and $\Gamma_{0}$, different two-dimensional current fields of vortex can be obtained according to Equation (11).

**Figure 2:**
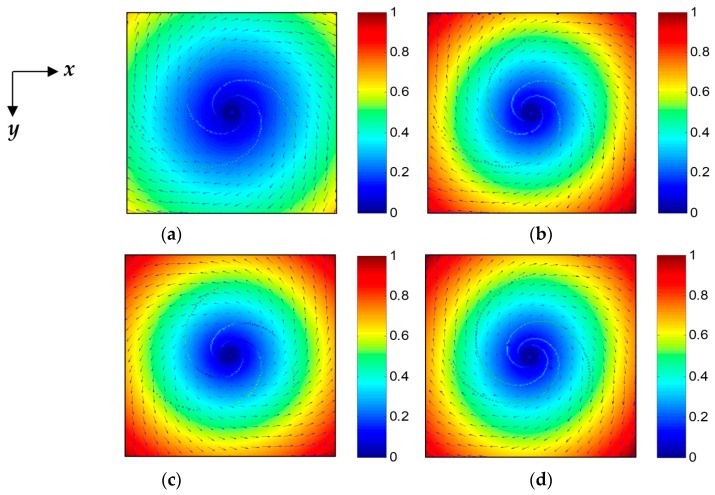
Simulated current fields of vortex based on the Burgers-Rott model. (**a**) α=0.0204, $\Gamma_{0}/\upsilon =10\pi$, the maximum velocity of the vortex current field $V_{\max}=0.68\;m/s$, and the rotation direction of the vortex current field is clockwise. (**b**) α=0.0272, $\Gamma_{0}/\upsilon =10\pi$, $V_{\max}=1\;m/s$, and the current field is clockwise. (**c**) α=−0.0272, $\Gamma_{0}/\upsilon =10\pi$, $V_{\max}=1\;m/s$, and the current field is anticlockwise. (**d**) α=0.0272, $\Gamma_{0}/\upsilon =12\pi$, $V_{\max}=1\;m/s$, and the current field is clockwise.

Figure 2 shows the simulated current fields of vortex. The parameters of the simulation are given in Table 1. Comparing Figure 2a,b, the value of α clearly affects the velocity magnitude of the vortex current field; the velocity magnitude increases with increasing α values. Moreover, the sign of α determines the rotation direction of the vortex current field. The rotation direction is clockwise when α is positive, but anticlockwise when α is negative, as shown in Figure 2b,c. The value of $\Gamma_{0}/\upsilon$ affects the shape of the vortex; the larger the value of $\Gamma_{0}/\upsilon$, the larger the curvature of the vortex, as shown in Figure 2b,d.

### 2.2. SAR Image Simulation

After the establishment of the eddy current field, an oceanic SAR imaging simulation model was required for simulating SAR eddy images. For this purpose, we selected M4S,f which was developed by Roland Romeiser of the University of Hamburg, Germany. M4S is a software toolkit based on a modified composite surface model for numerical simulations of SAR imaging of oceanic surface current features [24,25,26], and it can simulate surface wave spectra modulated by spatially varying currents. Different wind fields, radar and platform parameters can be set to investigate their impact on SAR eddy features with the same ocean surface current field induced by eddies.

**Figure 3:**
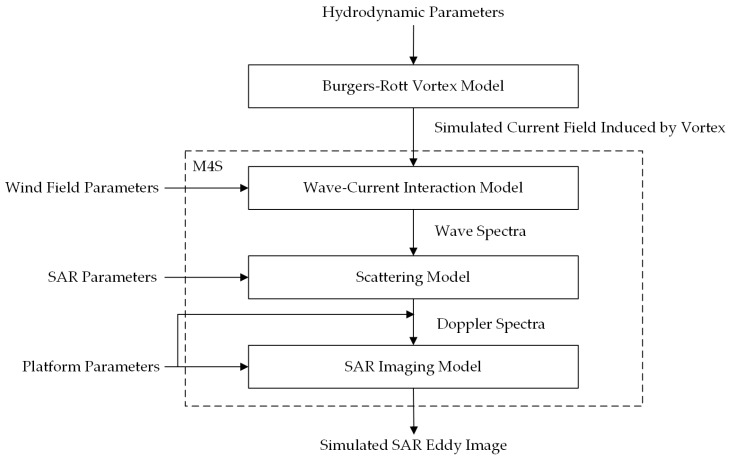
Flow chart of the simulation method.

**Figure 4:**
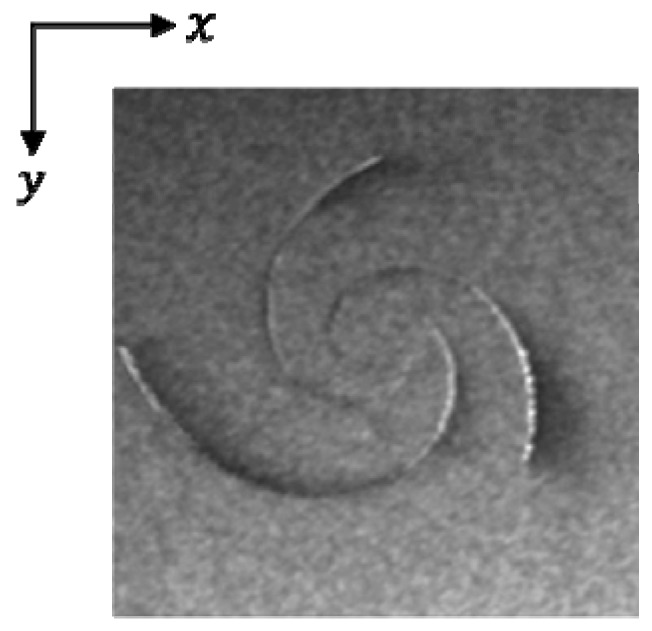
Simulated SAR image of an eddy. The size of the scene is 20 km × 20 km.

A flow chart of the simulation process is presented in Figure 3. M4S calculates the NRCS of the sea surface under given radar parameters by reading the sea surface wind field and current field as input data. Among them, the hydrodynamic parameters include α and $\Gamma_{0}/\upsilon$. The wind field parameters contain wind speed and direction. The SAR parameters include radar frequency, incidence angle, polarization, and look direction. The platform parameters are flight height and velocity. With the input current field established by the Burgers-Rott vortex model, wind field, and radar parameters, a simulated SAR eddy image is shown in Figure 4. The size of the scene is 20 km × 20 km, and the platform and radar parameters are given in Table 2. The hydrodynamic parameter α and $\Gamma_{0}/\upsilon$ was set to be −0.001486 and 10π, respectively. The wind speed was 5 m/s, and the wind direction was 225°.

## 3. Validation of the Simulation Method

In this section, simulated eddy images were compared with actual SAR images to validate the rationality of the proposed simulation method.

### 3.1. Example 1: The Experimental Validation Using ENVISAT-1 ASAR Image

**Figure 5:**
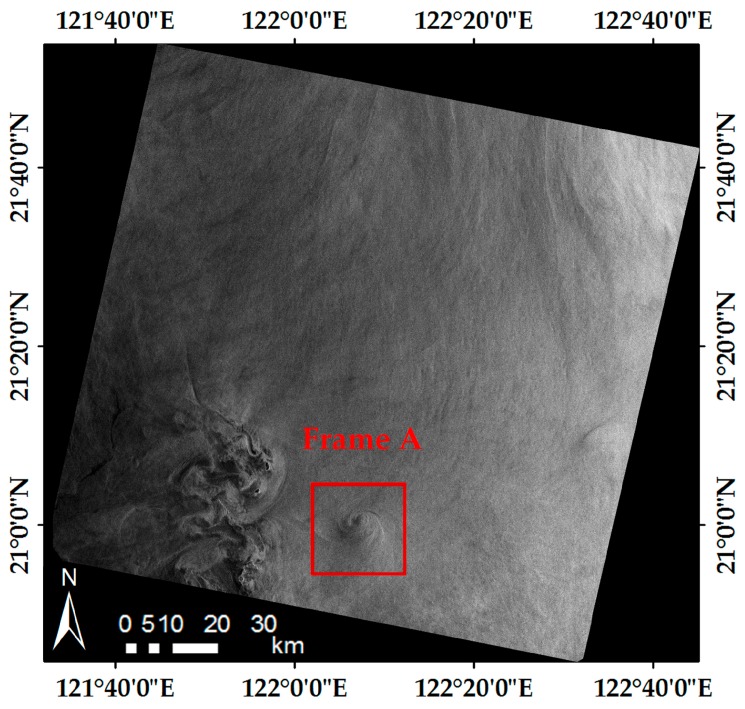
ENVISAT-1 ASAR image of the Luson Strait acquired on 27 April 2005 at 01:53:42 UTC. Frame A (20 km × 20 km) highlights a shear-wave-generated eddy.

**Figure 6:**
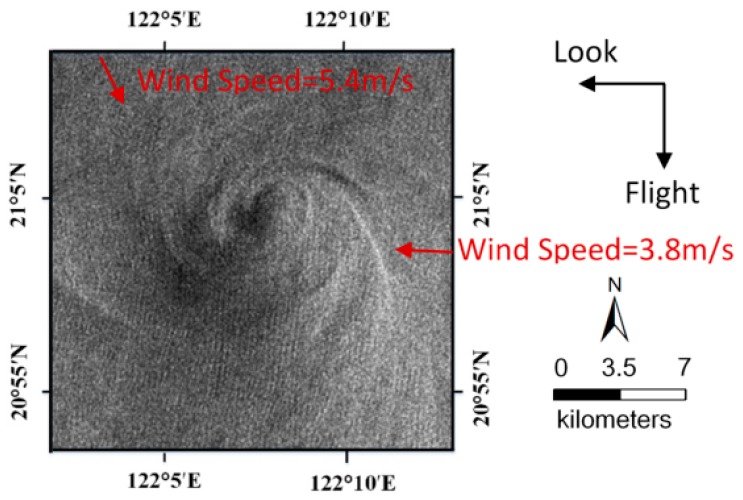
Enlargement of the shear-wave-generated eddy in Frame A. The flight and look direction of ENVISAT-1 ASAR are indicated by black arrows. Two wind vectors are shown as red arrows.

Figure 5 is an ENVISAT-1 ASAR IM image acquired in Luson Strait, on 27 April 2005 at 01:53:42 UTC. The related radar parameters are listed in Table 3. Frame A (20 km × 20 km) highlights a shear-wave-generated eddy with a diameter of about 11.2 km, which is shown in detail in Figure 6.

In Figure 6, the flight and look direction of ENVISAT-1 ASAR are indicated by black arrows. Two wind vectors were identified and shown as red arrows. The sea surface wind data was obtained from QuikSCAT on 27 April 2005 [27]. The grid resolution of the wind field is 25 km × 25 km over the ocean surface. The wind speeds and directions from left to right are listed as follows: (a) 5.44 m/s, 159.5° and (b) 3.80 m/s, 273.6°, where the wind direction is defined as the angle clockwise from the north in degrees. In addition, the corresponding current field reanalysis data was obtained from the Global Ocean Data Assimilation System (GODAS) with a spatial resolution of (1/3)° × 1°. A five-day average of the current field data from 25 April 2005 to 29 April 2005 was considered.

According to the above data, the wind speed near the eddy area is 5.21 m/s, the wind direction is 223.6°, and the current velocity is 0.26 m/s. Therefore, α was set to be −0.001486 according to Equation (11). $\Gamma_{0}/\upsilon$ was set to be 10π, adjusting the simulated eddy spirals to fit well with the actual eddy shape. The radar parameters were set with reference to the ENVISAT-1 ASAR parameters listed in Table 3. The size of the current field is 20 km×20 km, and the spatial resolution is 100 m × 100 m.

**Figure 7:**
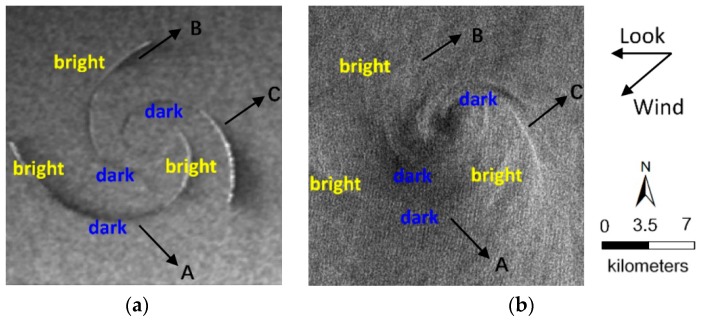
Comparison of (**a**) the simulated SAR eddy image and (**b**) the ENVISAT-1 ASAR image under the same radar parameters and wind field conditions. The radar look and wind direction are indicated by black arrows.

The comparison between the simulated and ENVISAT-1 ASAR image is illustrated in Figure 7a,b. The radar look and wind direction are indicated by black arrows. The eddy shape and brightness variations along eddy spirals in the simulated image appear to be consistent with the ENVISAT-1 ASAR image. Under the counterclockwise direction (the cyclonic eddy in the northern hemisphere rotates counterclockwise), the spirals show brightness variations from the outside to inside. The brightness variations along the longest spiral line A follow the order bright-dark-bright-dark, while spiral line B and C follows bright-dark. Such features are explained by changes in the spectral density of Bragg waves responsible for the backscattering of radar signals [28]. In addition, brightness variations along eddy spirals are periodic. A change from bright to dark can be defined as one alternation cycle, and the alternation cycle is related to the scale of the spirals, i.e., the alternation cycle increases as the spirals become longer.

### 3.2. Example 2: The Experimental Validation Using ERS-2 SAR Image

**Figure 8:**
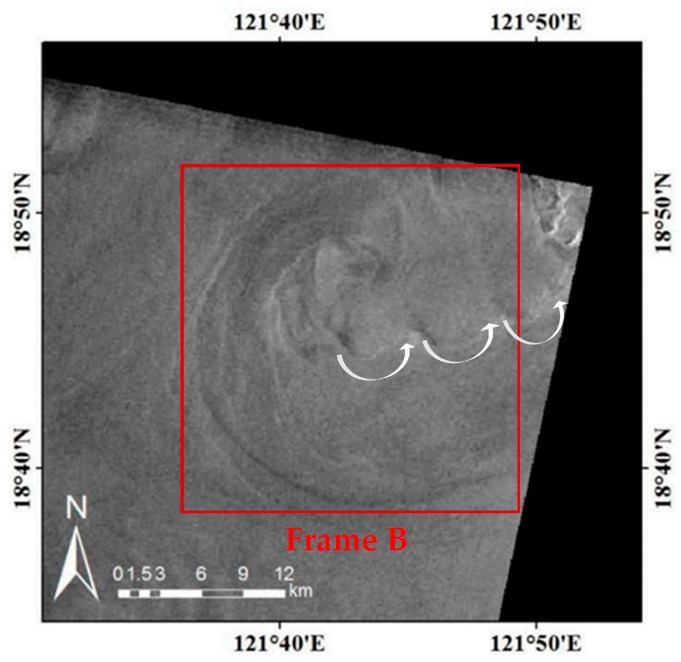
ERS-2 SAR image of the Luson Strait acquired on 11 June 2010 at 01:25:47 UTC. Frame B (24 km × 24 km) highlights a shear-wave-generated eddy.

**Figure 9:**
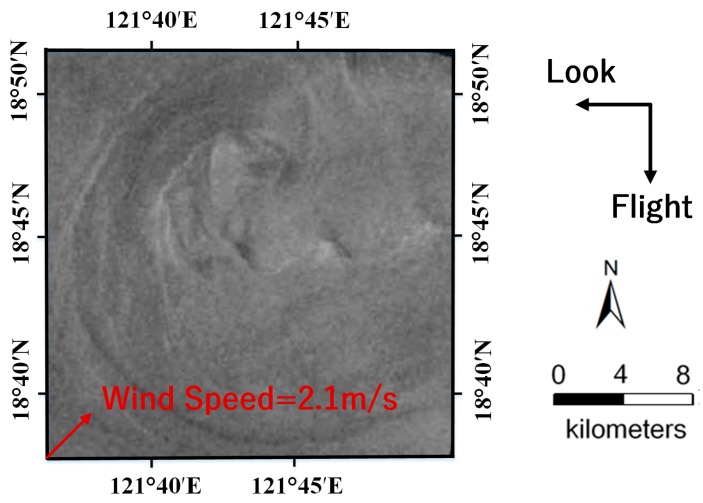
Enlargement of the shear-wave-generated eddy in Frame B. The flight and look direction of ERS-2 SAR are indicated by black arrows. The wind vector is shown as a red arrow.

To further verify the feasibility of the method, we performed a similar simulation experiment of an ERS-2 SAR image. Figure 8 is an ERS-2 SAR image acquired in the Luson Strait, on 11 June 2010 at 01:25:47 UTC. The related radar parameters are listed in Table 4. Frame B (24 km × 24 km) highlights a shear-wave-generated eddy with a diameter of about 24 km, which is shown in detail in Figure 9. In Figure 8, oceanic wakes generated by the island in the upper right corner of the image also exist, but this is beyond the scope of this paper.

In Figure 9, the flight and look direction of ERS-2 SAR are indicated by black arrows. The wind vector was identified and shown as a red arrow. The sea surface wind field reanalysis data was obtained from the Europe Centre for Medium-Range Weather Forecasts (ECMWF) on 11 June 2010. The grid resolution of the wind field is 0.125° × 0.125° over the ocean surface. In addition, the corresponding current field reanalysis data was obtained from GODAS with a spatial resolution of (1/3)° × 1°. A five-day average of the current field data from 10 June 2010 to 14 June 2010 was considered. According to the above data, the wind speed near the eddy area is 2.1 m/s, the wind direction is 45°, and the current velocity is 0.23 m/s. Therefore, α was set to be 0.000657 according to Equation (11). The radar parameters were set in reference to the ERS-2 SAR parameters listed in Table 4. The size of the current field is 24 km × 24 km, and the spatial resolution is 100 m × 100 m.

**Figure 10:**
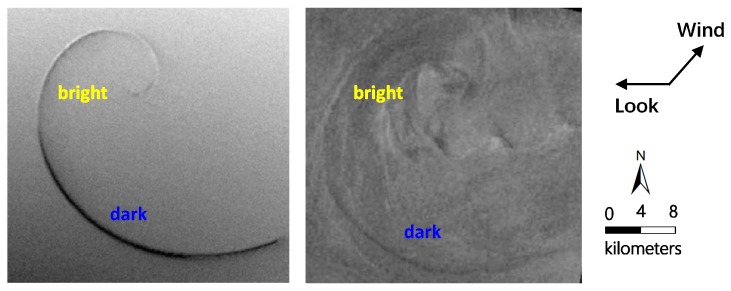
Comparison of (**a**) the simulated SAR eddy image and (**b**) the ERS-2 SAR image under the same radar parameters and wind field conditions. The radar look and wind direction are indicated by black arrows.

The comparison between the simulated and ERS-2 SAR image is illustrated in Figure 10a,b. The radar look and wind direction are indicated by black arrows. The island wakes are omitted during the simulation since we only focused on the eddy spiral. The eddy shape and brightness variations along the eddy spiral in the simulated image appear to be consistent with the ERS-2 SAR image. Under the clockwise direction (the anticyclonic eddy in the northern hemisphere rotates clockwise), the brightness variations along the spiral from the outside to the inside follow the order dark-bright. This result is consistent with the imaging geometry for an idealized anticyclonic eddy in Figure 1. Figure 10a shows the opposite of brightness variations along the eddy spiral, compared to Figure 1, since their radar look directions are opposite.

According to the above experimental validations, the proposed simulation method can realize an SAR image simulation of shear-wave-generated eddies. Nevertheless, some differences still exist between the simulated and actual SAR images. One of the most distinctive differences is around the eddy cores, where the NRCS of the simulated eddy should be darker. We believe that this is due to the incapability of the M4S model to take a three-dimensional current field as input. The altitude change caused by the eddy will generate a vertical velocity component, and it gets larger near the eddy core. Though the Burgers-Rott vortex model can generate a three-dimensional current field, M4S can only recognize the plane components, thus causing the inadequate modulation of NRCS around the eddy cores.

Some fine structures of the eddies cannot be simulated, although the agreement between the observed and simulated brightness variations of the eddy spirals is generally good. The radar imaging model used for this study is still based on a number of simplifying assumptions. For example, it is not clear whether the actual effect of a spatially varying atmospheric stratification on the surface wave field is always adequately represented by the effect of the proposed equivalent variations of $V_{x}$ and $V_{y}$. Furthermore, our surface wave model does not yet account for effects like wave breaking [29] or feedback between the surface roughness and wind stress [30]. The inclusion of such effects may lead to changes in the simulated radar signatures. Nevertheless, our proposed method is mainly focused on the features along the eddy spirals, since fully simulating the eddy features is too complicated; besides, eddy spirals are usually the only features which are detectable from actual SAR images. Our conclusions of the eddy spiral features may facilitate eddy detection, such as supervised or semi-supervised machine learning of shear-wave-generated eddy detection. We believe that the main results of this study are quite robust and not very sensitive to future model modifications.

## 4. Influence of Radar Look Direction and Wind Field on SAR Eddy Imaging

In this section, the images under different radar look directions, wind directions and wind speeds were generated by the proposed simulation method to analyze their influence on SAR eddy imaging.

Brightness varies not only along the eddy spirals but also throughout the SAR eddy image. Such brightness variations are, as a matter of fact, a modulation of the normalized radar cross section, which is also referred to as NRCS. The eddy spiral presents itself due to its higher or lower NRCS than the background. Along an eddy spiral, whether it gets brighter or darker, can be quantified as the NRCS contrast between values along the spiral and surrounding it. Therefore, Δσ is defined as the maximum NRCS contrast, or in other words, it is calculated from the brightest or darkest part of the eddy spiral to represent its visibility from the simulated image. The larger the value of Δσ, the clearer the spirals. To avoid the bias that may result from speckles or thermal noises, an average NRCS of twenty pixels was used for each along or beside the spiral to calculate the difference. In addition, the NRCS dynamic range of the background can also affect the visibility of the eddy, so $\Delta \sigma_{r}$ is defined as the NRCS contrast of the entire background image; it is also calculated from twenty pixels on average similar to Δσ. The larger the value of $\Delta \sigma_{r}$, the larger the NRCS contrast of the overall SAR image. Fifty simulations under each radar frequency have been conducted; the eddy spiral features and the calculated Δσ and $\Delta \sigma_{r}$ are almost the same.

In general, the influence of the radar look direction, wind direction and wind speed on SAR eddy imaging will be analyzed from four aspects: (1) the brightness variation along eddy spirals and (2) the brightness variation of the SAR image, which can be directly distinguished from simulated images; (3) the visibility of eddy spirals and (4) the brightness contrast of the SAR image, which can be quantified using Δσ and $\Delta \sigma_{r}$ respectively.

### 4.1. Influence of Radar Look Direction

**Figure 11:**
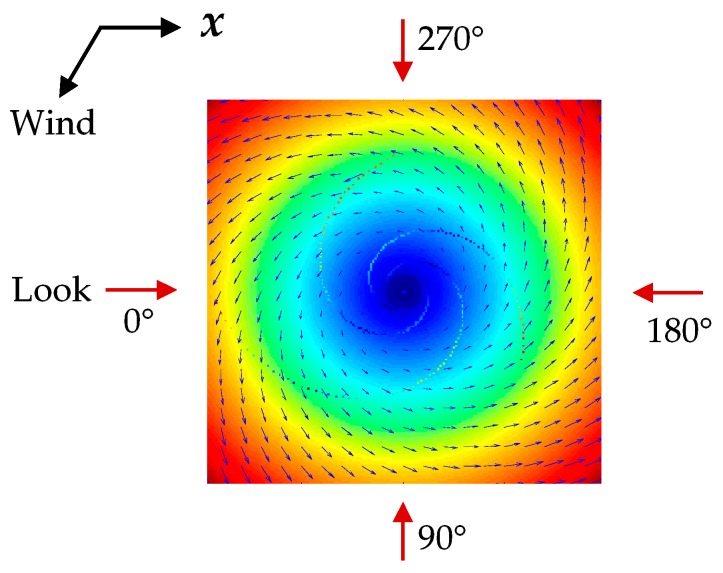
Four radar look directions with respect to the given current field. The red arrows represent the four radar look directions. The ***x*** axis of the current field and wind direction are indicated by black arrows.

**Figure 12:**
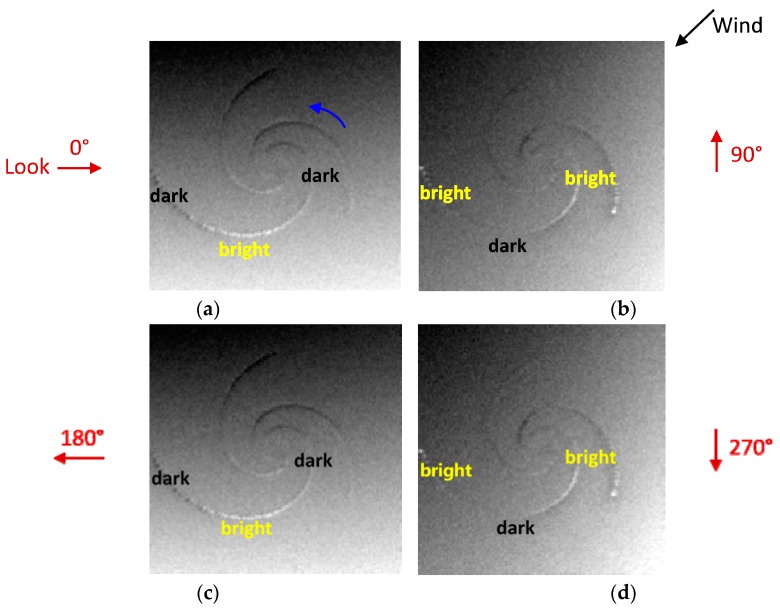
Simulated SAR eddy images under different radar look directions. (**a**–**d**) correspond to the look directions of 0°, 90°, 180°, and 270° respectively. The black arrow represents the wind direction and the blue arrow indicates the rotation direction of the current field.

During the simulations, the radar look directions are defined as the angle counterclockwise from the ***x*** axis of the current field in degrees, and they were selected as 0°, 90°, 180°, and 270° respectively. The four look directions with respect to the given current field are shown in Figure 11. The red arrows represent radar look directions. The ***x*** axis of the current field and wind direction are indicated by black arrows. Simulated SAR images under the four look directions are given in Figure 12. The parameters of the simulations are shown in Table 5.

In Figure 12, the red arrows represent the four different look directions, the black arrow represents the wind direction and the blue arrow indicates that the rotation direction of the eddy current field is counterclockwise. As shown in Figure 12a–d, the eddy spirals present different brightness variations, which are obviously related to the radar look directions. Under the look directions of 0° and 180°, the brightness variations are both dark-bright-dark from the outside to the inside of the eddy spirals. Meanwhile, under the look directions of 90° and 270°, the brightness variations are both bright-dark-bright. This indicates that the brightness variations along the eddy spirals are the same in two parallel look directions. Meanwhile, under two orthogonal look directions, the brightness variations are opposite. In addition, the results show that the simulated SAR images obtained under each look direction are darker in the upper left portion, but brighter in the lower right portion, which is due to the influence of the wind direction, as described in Section 4.2.

**Figure 13:**
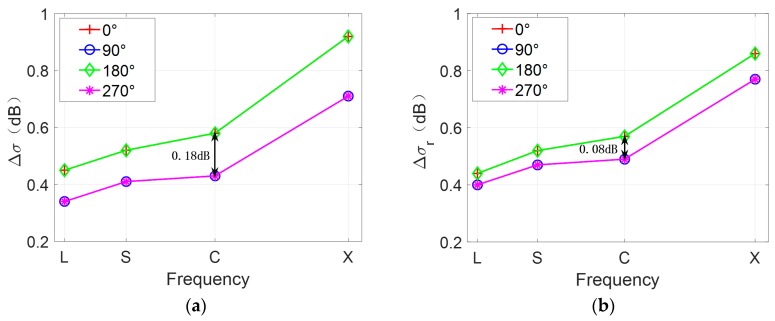
(**a**) Normalized radar cross section (NRCS) contrast of eddy spirals Δσ and (**b**) NRCS contrast of SAR image $\Delta \sigma_{r}$ under different look directions and radar frequencies. Fifty simulations are averaged to reduce speckle bias.

In general, the radar look direction determines the brightness variations along the eddy spirals, but it has a limited effect on the brightness variations of the SAR image. To further analyze the influence of the radar look direction on SAR eddy imaging, Δσ and $\Delta \sigma_{r}$ of Figure 12a–d were calculated. The results are shown in Figure 13. To verify the validity of this result under different radar frequencies, L-, S-, and X-band were also considered.

As shown in Figure 13a, the values of Δσ are the same in two parallel look directions, such as 0° and 180°, which indicates the same visibility of eddy spirals under two parallel look directions. Moreover, Figure 13a,b shows that there is a considerable value difference of Δσ between two orthogonal look directions; however, the value difference of $\Delta \sigma_{r}$ is relatively small. For example, under the look direction of 0° and 180° at C-band, the value difference of Δσ is about 0.18 dB, while the value difference of $\Delta \sigma_{r}$ is only 0.08 dB. This indicates that the radar look direction has more influence on the visibility of eddy spirals than the brightness contrast of the overall SAR image. In addition, when the look direction is 0° or 180°, the values of Δσ are larger, which means that the eddy is relatively more obvious and conducive to be observed by SAR in this condition. On the other hand, as the radar frequency increases, the values of Δσ and $\Delta \sigma_{r}$ increase gradually. It is apparent that the eddy features in the SAR images become clearer at higher radar frequencies.

In summary, the radar look direction mainly affects brightness variations and the visibility of eddy spirals. The simulation results of Figure 12a are consistent with the imaging geometry for the idealized anticyclonic eddy in Figure 1. When the radar look direction is 0° and the current field direction is counterclockwise, the longest spiral of the cyclonic eddy changes from dark to bright and then to dark, which is opposite to the brightness variations of the idealized anticyclonic eddy. Furthermore, the conclusion of two orthogonal look directions is in accordance with the analysis of an ERS-1 SAR eddy image in Ref. [11], thus verifying the effectiveness of the simulation.

### 4.2. Influence of Wind Direction

**Figure 14:**
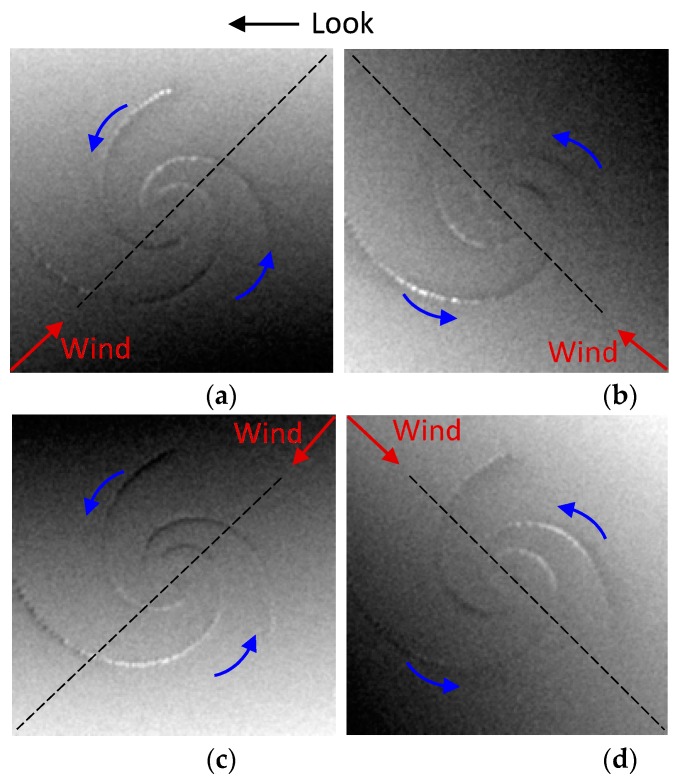
Simulated SAR images under different wind directions. (**a**–**d**) correspond to the wind directions of 45°, 135°, 225°, and 315° respectively. The black arrow represents the radar look direction and the blue arrow indicates the rotation direction of the current field.

During the simulations, the wind directions are defined as the angle counterclockwise from the ***x*** axis of the current field in degrees and were selected as 45°, 135°, 225°, and 315° respectively. The radar look direction was 180°, and the other parameters were the same as those described in Section 4.1, as shown in Table 6. The simulated SAR images under the four different wind directions are given in Figure 14.

**Figure 15:**
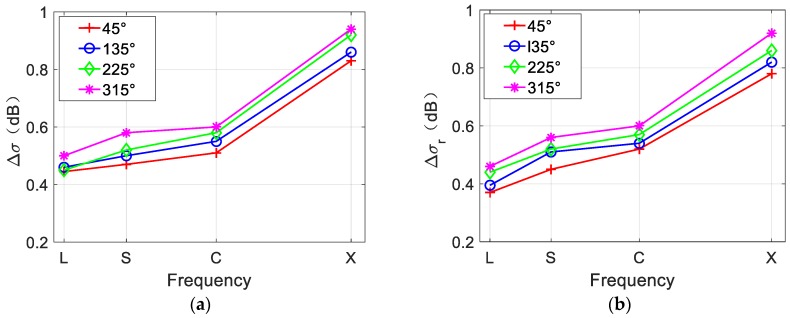
(**a**) NRCS contrast of eddy spirals Δσ and (**b**) NRCS contrast of SAR image $\Delta \sigma_{r}$ under different wind directions and radar frequencies. Fifty simulations are averaged to reduce speckle bias.

In Figure 14, the red arrows represent four different wind directions, the black arrow represents the radar look direction, and the blue arrows indicate that the eddy current field rotates in the counterclockwise direction. Each simulated SAR image is divided into two parts by its diagonal. As shown in Figure 14a–d, the brightness varies across the image and the brightness variation is obviously related to the wind directions, that is, half of the image with the current field direction opposite to the wind direction is brighter, whereas the other half is darker. This phenomenon can also be observed in Figure 7. The lower right portions of Figure 7a,b are brighter than the other area is. This indicates that the wind direction determines the brightness variations of the overall SAR image. Though the brightness variations along the eddy spirals is different under different wind direction, it is merely caused by the brightness variation of the entire SAR image, since when half of the image is brighter, so are the eddy spirals in it. To further analyze the influence of wind direction on SAR eddy imaging, Δσ and $\Delta \sigma_{r}$ of Figure 14a–d were calculated. The results are shown in Figure 15. L-, S-, and X-band were also considered.

Figure 15a,b shows that under different wind directions, the values of Δσ and $\Delta \sigma_{r}$ are slightly different. This implies that wind direction has minor effect on the visibility of eddy spirals and the brightness contrast of the overall SAR image. Therefore, wind direction generally affects the brightness variations of the SAR image, and half of the image with the current field direction opposite to the wind direction is brighter, whereas the other half is darker. In addition, the same conclusion as in Figure 13 can be obtained. The values of Δσ and $\Delta \sigma_{r}$ increase as the radar frequency increases.

### 4.3. Influence of Wind Speed

**Figure 16:**
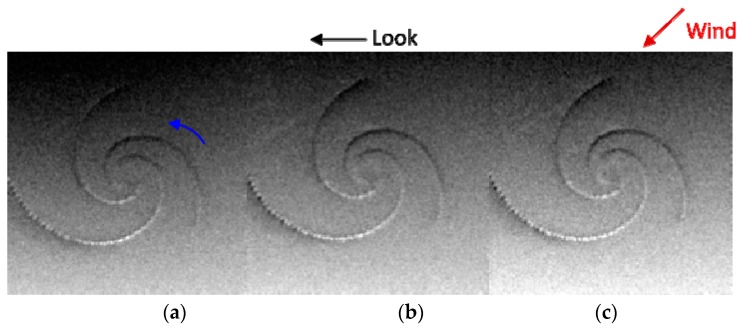
Simulated SAR images under different wind speeds. (**a**–**c**) correspond to wind speeds of 4 m/s, 7 m/s, and 10 m/s respectively. The red arrow represents the wind direction, the black arrow represents the radar look direction, and the blue arrow indicates the rotation direction of the current field.

To analyze the influence of wind speed on SAR eddy imaging, the radar look direction and wind direction were kept constant, and wind speeds were set to be 4 m/s, 7 m/s and 10 m/s respectively. The other parameters of simulations are given in Table 7. The simulated SAR images under different wind speeds are shown in Figure 16.

**Figure 17:**
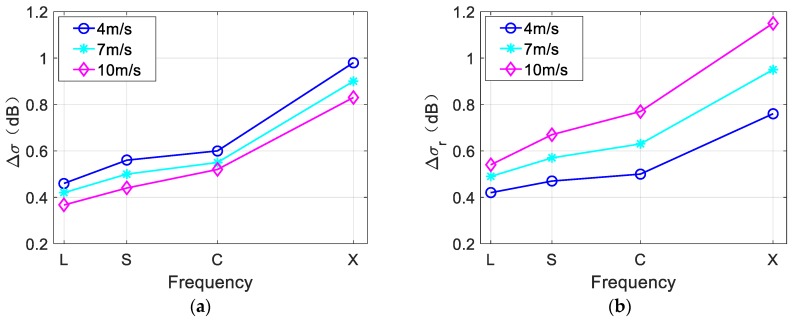
(**a**) NRCS contrast of eddy spirals Δσ and (**b**) NRCS contrast of SAR image $\Delta \sigma_{r}$ under different wind speeds and radar frequencies. Fifty simulations are averaged to reduce speckle bias.

In Figure 16, the red arrow represents the wind direction, the black arrow represents the radar look direction, and the blue arrow indicates that the eddy current field rotates in the counterclockwise direction. As shown in Figure 16a–c, wind speed does not change the brightness variations along the eddy spirals, but affects the brightness variations of the overall SAR image. With increases in wind speed, the entire simulated image becomes brighter. To further analyze the influence of wind speed on SAR eddy imaging, Δσ and $\Delta \sigma_{r}$ of Figure 16 were calculated. The results are shown in Figure 17. L-, S-, and X-band were also considered.

In Figure 17a, with increasing wind speed, the value of Δσ becomes smaller, indicating that the eddy spirals become less obvious. Meanwhile, in Figure 17b, the value of $\Delta \sigma_{r}$ increases with the increasing wind speed, indicating that the brightness contrast of the entire SAR image increases. In addition, there is a considerable value difference of $\Delta \sigma_{r}$ between different wind speeds, but the value difference of Δσ is relatively small. This suggest that the wind speed has more influence on the brightness contrast of the overall SAR image than on the visibility of the eddy spirals.

On the other hand, the results indicate that the values of Δσ and $\Delta \sigma_{r}$ are larger at higher radar frequencies, which is the same as the conclusion drawn from Figure 13 and Figure 15. Referring to existing theories, a possible explanation for this conclusion should be the less defocusing effect as the radar frequency gets higher. According to SAR imaging theory, the high resolution along the flight direction is realized by synthesizing a large virtual aperture within the synthetic aperture time. However, the best resolution along the flight direction is restricted by its actual antenna aperture length, which is half of the antenna aperture. To achieve the best resolution, the synthetic aperture time should be [31]:

(12) $$T=0.886\frac{cR}{DV_{SAR}f}$$

where f is the radar frequency, c is the speed of light, R is the nearest range between the platform and imaged target, $V_{SAR}$ is the platform velocity, and D is the actual antenna aperture. The synthetic aperture time, as a matter of fact, is the integrating time for the backscattered energy of a target to be well focused. For the same set of antennae, a longer integrating time is needed for a higher radar frequency, according to Equation (12). However, within the integrating time, moving targets will get defocused, and this is inevitable especially when imaging the ocean surface. Therefore, under a higher radar frequency, the SAR image of the eddy suffers less from defocusing due to a shorter integrating time, the spirals are more obvious and the brightness contrast is larger. However, many additional multi-frequency radar images of shear-wave-generated eddies will be needed for a validation of this conclusion, in our opinion. This issue needs to be addressed in more detail in future projects and experiments. We think that an important and solid conclusion can be drawn from our results, despite some unresolved theoretical problems.

## 5. Conclusions

Based on the Burgers-Rott vortex model, a SAR image simulation method for oceanic shear-wave-generated eddies is proposed in this paper. Furthermore, comparative analyses have proven that the simulated images correspond well to the actual SAR images.

The simulated SAR images indicate that eddy spirals exhibit brightness variations, and the alternation cycles of brightness variations are related to the scale of eddy spirals. However, the quantitative relationship between the alternation cycles and the scale of spirals still needs to be resolved through further statistical comparisons between the simulated and actual SAR images.

SAR images simulated under different radar look directions show that the look direction mainly affects the SAR imaging of eddy spirals. The brightness variations along eddy spirals remain the same under two parallel look directions but show opposite trends under two orthogonal look directions. The visibility of eddy spirals under two parallel look directions is also the same and the spirals are more obvious under radar look directions of 0° or 180°. SAR images simulated under different wind fields show that wind direction and wind speed mainly affect the SAR imaging of the whole eddy area. Wind direction affects the brightness variations throughout the SAR image, and half of the image with the current field direction opposite to the wind direction is brighter, whereas the other half is darker. Wind speed affects the brightness variations and the brightness contrast of the SAR image. With an increased wind speed, the image is brighter and its brightness contrast is higher. Therefore, in future SAR observations of eddies, brightness features due to different radar look directions and wind field should be considered while interpreting eddy images. Moreover, radars at higher frequencies also facilitate the observation of eddy features. To our knowledge, a comparable agreement between observed and simulated radar signatures of the shear-wave-generated eddies at more than one frequency, look direction and wind field has never been conducted in previous studies.

SAR imaging of oceanic eddies is affected by radar parameters and environmental factors, and the simulation method proposed in this paper can facilitate research on eddy features by changing radar parameters and environmental conditions. The simulation results can interpret and evaluate the effects of radar look direction and wind field conditions on SAR eddy imaging and provide guidance for interpreting eddy features in SAR images. Nevertheless, the proposed simulation method and results in this context are mainly focused on shear-wave-generated eddies. Other SAR imaging mechanisms of oceanic eddies, including film mechanisms, thermal mechanisms, and ice mechanisms, need to be resolved through further research.

## Figures and Tables

**Table 1:** The simulation parameters in Figure 2.

Figure	*α*	Γ_0_/υ	*V*_max_	Rotation Direction
(a)	0.0204	10 π	0.68 m/s	clockwise
(b)	0.0272	10 π	1 m/s	clockwise
(c)	−0.0272	10 π	1 m/s	anticlockwise
(d)	0.0272	12 π	1 m/s	clockwise

**Table 2:** The simulation parameters of Figure 4.

Parameters	Values
Radar look direction	180°
Incidence angle	23°
Radar frequency	C-band
Polarization	HH
Platform height	800 km
Platform velocity	7455 m/s

**Table 3:** The related parameters of ENVISAT-1 ASAR.

Platform	Polarization	Band	Boresight Incidence Angle	Platform Height	Platform Velocity
ENVISAT-1	HH	C	23.1°	800 km	7455

**Table 4:** The related parameters of ERS-2 Synthetic Aperture Radar (SAR).

Platform	Polarization	Band	Boresight Incidence Angle	Platform Height	Platform Velocity
ERS-2	VV	C	23°	780 km	7500 m/s

**Table 5:** The simulation parameters of Figure 12.

Parameters	Values
Radar look direction	0°, 90°, 180°, 270°
Incidence angle	25°
Radar frequency	C-band
Polarization	HH
Platform height	800 km
Platform velocity	7455 m/s
Wind direction	225°
Wind speed	6 m/s
Rotation direction of current field	counterclockwise
Maximum Current velocity	1 m/s

**Table 6:** The simulation parameters of Figure 14.

Parameters	Values
Radar look direction	180°
Incidence angle	25°
Radar frequency	C-band
Polarization	HH
Platform height	800 km
Platform velocity	7455 m/s
Wind direction	45°, 135°, 225°, 315°
Wind speed	6 m/s
Rotation direction of current field	counterclockwise
Maximum Current velocity	1 m/s

**Table 7:** The simulation parameters of Figure 16.

Parameters	Values
Radar look direction	180°
Incidence angle	25°
Radar frequency	C-band
Polarization	HH
Platform height	800 km
Platform velocity	7455 m/s
Wind direction	225°
Wind speed	4 m/s, 7 m/s, 10 m/s
Rotation direction of current field	counterclockwise
Maximum Current velocity	1 m/s

## References

[1] Karimova S. An Approach to Automated Spiral Eddy Detection in Sar Images. Proceedings of the Igarss IEEE International Geoscience & Remote Sensing Symposium.

[2] Dreschler-Fischer L., Lavrova O., Seppke B., Gade M., Bocharova T., Serebryany A., Bestmann O. Detecting and Tracking Small Scale Eddies in the Black Sea and the Baltic Sea using High-Resolution Radarsat-2 and Terrasar-x Imagery (dteddie). Proceedings of the Geoscience and Remote Sensing Symposium.

[3] Johannessen J.A., R∅ed L.P., Wahl T. (2012). Eddies detected in ERS-1 SAR images and simulated in reduced gravity model. Int. J. Remote Sens..

[4] Schuler D.L., Lee J.S., Grandi G.D. Spiral Eddy Detection Using Surfactant Slick Patterns and Polarimetric Sar Image Decomposition Techniques. Proceedings of the IEEE International Geoscience & Remote Sensing Symposium.

[5] Liu A.K., Peng C.Y., Schumacher J.D. (1994). Wave-current interaction study in the gulf of alaska for detection of eddies by synthetic aperture radar. J. Geophys. Res. Oceans.

[6] Karimova S., Gade M. (2016). Improved statistics of sub-mesoscale eddies in the baltic sea retrieved from SAR imagery. Int. J. Remote Sens..

[7] Xu G., Yang J., Dong C., Chen D., Wang J. (2015). Statistical study of submesoscale eddies identified from synthetic aperture radar images in the luzon strait and adjacent seas. Int. J. Remote Sens..

[8] Karimova S. (2012). Spiral eddies in the baltic, black and caspian seas as seen by satellite radar data. Adv. Space Res..

[9] Karimova S.S., Lavrova O.Y., Solov’Ev D.M. (2012). Observation of eddy structures in the baltic sea with the use of radiolocation and radiometric satellite data. Izv. Atmos. Ocean. Phys..

[10] Ivanov A.Y., Ginzburg A.I. (2002). Oceanic eddies in synthetic aperture radar images. J. Earth Syst. Sci..

[11] Lyzenga D., Wackerman C. Detection and classification of ocean eddies using ERS-1 and aircraft SAR images. Proceedings of the Third ERS Symposium on Space at the service of our Environment.

[12] Johannessen J.A., Shuchman R.A., Digranes G., Lyzenga D.R., Wackerman C., Johannessen O.M., Vachon P.W. (1996). Coastal ocean fronts and eddies imaged with ERS 1 synthetic aperture radar. J. Geophys. Res. Oceans.

[13] Alpers W., Brandt P., Lazar A., Dagorne D., Sow B., Faye S., Hansen M., Rubino A., Poulain P.M., Bremer P. (2013). A small-scale oceanic eddy off the coast of West Africa studied by multi-sensor satellite and surface drifter data, and by a numerical model. Remote Sens. Environ..

[14] Friedman K.S., Li X., Pichel W.G., Clemente-Colon P. Eddy detection using radarsat-1 synthetic aperture radar. Proceedings of the Geoscience and Remote Sensing Symposium (IGARSS ’04).

[15] Mitnik L.M., Dubina V.A., Shevchenko G.V. Ers sar and envisat asar observations of oceanic dynamic phenomena in the southwestern okhotsk sea. Proceedings of the 004 Envisat & ERS Symposium.

[16] Cooper A.L., Shen C.Y., Marmorino G.O., Evans T. (2005). Simulated radar imagery of an ocean “spiral eddy”. IEEE Trans. Geosci. Remote Sens..

[17] Rankine W.J.M. (1872). A Manual of Applied Mechanics.

[18] Oseen C.W. (1911). Ber die Stoke’sche Formel und Über Eine Verwandte Aufgabe in der Hydrodynamik.

[19] Burgers J. (1940). Application of a Model System to Illustrate Some Points of the Statistical Theory of Free Turbulence.

[20] Burgers J.M. (1948). A mathematical model illustrating the theory of turbulence. Adv. Appl. Mech..

[21] Rott N. (1958). On the viscous core of a line vortex. Z. Angew. Math. Phys. ZAMP.

[22] Rott N. (1959). On the viscous core of a line vortex ii. Zeitschrift für Angewandte Mathematik und Physik.

[23] Sullivan R.D. (1959). A two-cell vortex solution of the navier-stokes equations. J. Aerosp. Sci..

[24] Romeiser R., Alpers W., Wismann V. (1997). An improved composite surface model for the radar backscattering cross section of the ocean surface: 1. Theory of the model and optimization/validation by scatterometer data. J. Geophys. Res. Oceans.

[25] Romeiser R., Alpers W. (1997). An improved composite surface model for the radar backscattering cross section of the ocean surface: 2. Model response to surface roughness variations and the radar imaging of underwater bottom topography. J. Geophys. Res. Oceans.

[26] Romeiser R., Thompson D.R. (2000). Numerical study on the along-track interferometric radar imaging mechanism of oceanic surface currents. IEEE Trans. Geosci. Remote Sens..

[27] Liu A.K., Hsu M.K. (2009). Deriving ocean surface drift using multiple SAR sensors. Remote Sens..

[28] Mitnik L., Dubina V., Lobanov V. Cold Season Features of the Japan Sea Coastal Zone Revealed by Ers Sar. Proceedings of the IEEE International Geoscience & Remote Sensing Symposium.

[29] Lyzenga D.R. Effects of Wave Breaking on Sar Signatures Observed Near the Edge of the Gulf Stream. Proceedings of the International Geoscience & Remote Sensing Symposium.

[30] Romeiser R., Schmidt A., Alpers W. (1994). A three-scale composite surface model for the ocean wave-radar modulation transfer function. J. Geophys. Res. Oceans.

[31] Gumming I.G., Wong F.H. (2005). Digital Processing of Synthetic Aperture Radar Data: Algorithms and Implementation.

